# Short-wave diathermy in the clinical management of musculoskeletal disorders: a pilot observational study

**DOI:** 10.1007/s00484-019-01806-x

**Published:** 2019-11-20

**Authors:** Stefano Masiero, Andrea Pignataro, Giovanni Piran, Miriam Duso, Patrice Mimche, Mario Ermani, Alessandra Del Felice

**Affiliations:** 1 Rehabilitation Unit, Department of Neuroscience, University of Padova, Via Giustiniani 2, 35128 Padova, Italy; 2 Padova Neuroscience Center (PNC), University of Padova, Via Orus 2/B, 35131 Padova, Italy; 3 Neurology Unit, Department of Neuroscience, University of Padova, Via Giustiniani 2, 35128 Padova, Italy

**Keywords:** Knee osteoarthritis, Neck/back pain, Tendinopathies, Electromagnetic radio waves, Physical therapies

## Abstract

Musculoskeletal disorders are the most common cause of pain and functional limitation in the general population. The study aim was  to evaluate short-wave diathermy (SWD) effects on pain and quality of life in people with musculoskeletal disorders. Eighty participants (31 men, mean age 56 ± 12.49 years) were enrolled, recruiting from outpatient clinics at the Rehabilitation Unit, University Hospital, Padova. Inclusion criteria were pain lasting more than 15 days, pain visual analog scale (VAS) score higher than 50/100 mm, and a diagnosis of osteoarthritis, neck/back pain, or tendinopathies. All participants underwent ten sessions of percutaneous SWD, 3 times/week. Each session lasted 15–20 min, with frequencies of 4 or 8 MHz and heat intensity between 40 and 60 W. Outcomes were assessed before and after treatment. Primary outcome was pain reduction, evaluated by short form McGill pain questionnaire, which includes VAS and present pain intensity (PPI). Secondary outcome was improvement in social and work-related activity limitations. Participants were grouped based on classification of pain [nociceptive and neuropathic pain (group A) vs nociceptive only (group B)]. VAS and PPI improved significantly (*p* < 0.01). No difference in pain reduction (VAS and PPI) emerged between the groups. Limitations due to pain in work-related and non-work-related activities decreased (*p* < 0.01); use of pain medications was reduced at T1 vs T0 (*p* < 0.01). Our results suggest that SWD is effective in reducing musculoskeletal pain in the short term, providing relief and improving quality of life.

## Introduction

Musculoskeletal disorders are the most common cause of pain, disability, and functional limitation (Yoshimura et al. 2009). Among them, low back pain (LBP) and osteoarthritis (Wang 2012; Hoy et al. 2014) are major determinants. A variety of physical therapies are available for pain treatment, such as low- and high-intensity laser therapy (Boyraz et al. 2015; Glazov et al. 2016), electrotherapy (Desmeules et al. 2016), and magnetotherapy (Kanat et al. 2013).

Heat therapy is commonly used. It can be divided into superficial and deep treatment. Superficial heat therapy, which includes hot packs and thermal water, acts through skin contact (Masiero et al. 2018). Deep heat therapy is generated by the interaction of electromagnetic waves with biological tissues (Laufer and Dar 2012). Different types of waves can be used in diathermy. Long-wave diathermy (LWD), short-wave diathermy (SWD), and microwave diathermy (MWD) are the most common. Long-wave (LW) frequencies are between 3 and 300 kHz, short waves (SW) between 3 and 30 MHz, and microwaves (MW) between 300 and 3000 GHz (“Classificazioni delle onde elettromagnetiche” n.d., www.peduto.it). SWD effects can be divided into thermal and not thermal. Thermal effects induce vasodilatation, elevation of pain threshold, reduction in muscle spasm, acceleration of cellular metabolism, and increased soft tissue extensibility (Kitchen et al. 1992; Shields et al. 2002). The athermal effects are likely the result of cell’s energy absorption from oscillating electrical fields (Laufer and Dar 2012), inducing or enhancing cellular activity. They include increased blood flow, decreased joint pain and stiffness, reduced inflammation, faster resolution of edema, and accelerated wound healing (Al-Mandeel and Watson 2010). Short-wave therapy can be delivered either in a continuous or a pulse mode (Wang et al. 2017).

Continuous short-wave diathermy (cSWD) is generally used for its thermal effects whereas pulsed short-wave diathermy (pSWD) for athermal effects. Recent studies have demonstrated that pSWD may also induce an elevation of tissue temperature that is dependent on the total average power delivered (Murray and Kitchen 2000). Doubts cast on the real effects of athermal phenomena (Laufer and Dar 2012) suggest that the clinical effects of SWD are mainly related to increased temperature. Reduction in pain is one of the most important effects of diathermy, although the physiological basis is poorly understood. Heating may reduce pain by promoting vasodilatation and efflux from the affected tissue of pain mediators, e.g., bradykinin, serotonin, and prostaglandins (Goats 1989). Another possible mechanism of action is the inhibition of nociceptive transmission by activation of A-alpha and A-beta fibers (Melzack et al. 1977) or by stimulation of the cutaneous thermoreceptors; this mechanism, known as gate control, blocks the transmission of pain as it enters the spinal cord (Goats 1989). In addition, muscle spasm due to musculoskeletal pain is often reduced by heat and this in turn can contribute to pain decrease (Goats 1989). SWD improves cellular healing processes, producing an overexpression of heat shock proteins (HSP), which contribute to intracellular protein repair. High levels of HSP increase the speed of healing of cells and tissues and may be important in preventing skeletal muscle breakdown during exercise (Terauchi et al. 2003; Costantino et al. 2005; McCarthy et al. 2006).

The real efficacy of SWD in the treatment of musculoskeletal disorders has not yet been fully clarified, with studies focusing mainly on pSWD for treatment of knee osteoarthritis (Laufer and Dar 2012; Wang et al. 2017) and scarce interest for other conditions, such as tendinopathies and low back pain. Lastly, the availability and use of cSWD decreased in contrast to pSWD, as the latter were considered free of thermal effects and thereby safer (Shah and Ghani 2007).

The aim of this study is to evaluate pain reduction in people with musculoskeletal disorders using continuous SWD at 4 or 8 MHz frequencies, according to a personalized administration protocol. The secondary outcome is the improvement in social and work-related activity limitations and reduction in use of pain medications.

## Materials and methods

### Study design

This is an observational longitudinal study. Only SWD treatment was allowed during the study. The therapeutic program with SWD consisted of ten percutaneous short-wave diathermy sessions, three times per week. Each session lasted 15–20 min and was performed by an expert physician, with specific training in device use. Overall, therapy lasted three and a half weeks. None of the participants underwent other forms of physical therapy or therapeutic exercise, nor were they allowed to take any painkiller during the period of treatment.

This study followed all the recommendations for research in human beings (World Medical Association 2013) and participants signed the informed consent form before being included, according to the institutional review board.

### Population

We enrolled persons referred to the outpatient Clinic of the Rehabilitation Unit of the University Hospital (Padova, Italy) for musculoskeletal pain from January 2016 to March 2017.

The inclusion criteria were as follows: (1) pain with VAS (visual analog scale, see below) value above 50/100 ml, (2) pain of musculoskeletal origin (knee osteoarthritis, neck/back pain, tendinopathies of shoulder and elbow), (3) pain lasting longer than 15 days, and (4) older than 18 years.

Exclusion criteria were as follows: (1) presence of a pacemaker, (2) pregnancy, (3) malignancy, and (4) older than 80 years.

Participants were classified as follows: group A, subjects with both nociceptive and neuropathic pain, defined as nociceptive pain (i.e., with a myofascial or tendon component) plus a neuropathic involvement (e.g., LBP with radicular irradiation); group B, subjects with only nociceptive pain. Presence of neuropathic pain was assessed with the Neuropathic Pain Symptom Inventory (Bouhassira et al. 2010). Neuropathic and nociceptive pain was defined as the presence of these two forms over the same anatomical/metameric distribution.

### Clinical examination

All participants were blindly evaluated by a physician different from the enrolling and treating one 1 week prior to starting the therapeutic program (T0) and 4 weeks after the last session (T1). Participants were assessed as follows:

1. Short form of McGill pain questionnaire (MPQ).
2. SF-MPQ, the main component of the SF-MPQ consists of 15 descriptors (11 sensory, 4 affective) which are rated on an intensity scale as 0 = none, 1 = mild, 2 = moderate, or 3 = severe. The SF-MPQ also includes the present pain intensity (PPI) index and a visual analog scale (VAS). In PPI, the subject is asked to mark his pain from 0 (no pain) to 5 (excruciating pain) (Melzack 1987). VAS consists of a straight line with the endpoints defining extreme limits, such as “no pain at all” and “pain as bad as it could be”. The participant is asked to mark his/her pain level on the line between the two endpoints 100 mm apart (Carlsson 1983). The distance between “no pain at all” and the mark then defines the subject’s pain over the last week.
3. Two items of short form-12 (SF-12) (Ware et al. 1996) standard index about work-related and non-work-related activity limitations were administered. The SF-12 is a short version of the short form-36 (SF-36), a measure of physical and mental health functioning that has been widely used and validated. Work-related and social activity questions investigate interference of pain with normal work (including work outside home and housework) and the impact of physical health or emotional problems interfered with social activities (like visiting friends and relatives) in the past 4 weeks, respectively. The two multiple-choice questions were the following:
  - “In the last four weeks, how much did pain interfere with your normal everyday tasks (including work inside and outside your home)? 1. Not at all; 2. Slightly; 3. Moderately; 4. Quite a lot; 5. Extremely” (Ware et al. 1996);
  - “During the last four weeks, how much time have your physical health or emotional problems interfered with your social activities (like visiting friends, relatives, etc.)? 1. All of the time; 2. Most of the time; 3. Some of the time; 4. A little of the time; 5. None of the time” (Ware et al. 1996).

### Procedure

A ProNexibus Plus-FocusMed® device was used to deploy SWD. It is equipped with a handpiece covered with titanium dioxide, a passive plate, and an impedance meter evaluating absorption. Before use, as requested by the current European legislation on medical devices and workplace safety, the short-wave electrometrical device was checked by a qualified medical engineer that verified conformity and CE marking.

Frequencies of 4 or 8 MHz (power 40 W and 60 W) were used, selected based on the affected region (i.e., lower frequencies for more superficial structures, i.e., superficial muscles or tendons, higher frequencies for joints, radicular involvement, deep muscles). Different frequencies could be delivered in sequence during the same session.

Participants were instructed to refer when thermal sensation was felt and to warn the physician when the sensation of heat exceeded the value of 6 (based on a scale of 0–10, where 0 is no heat sensation and 10 is maximum heat sensation). In that case, the operator reduced power output.

### Statistical analysis

*T*-test for independent groups was used for normal distribution variables. A non-parametric test was used for the evaluation of scores and their variations, being the scores ordinal qualitative variables. The Mann-Whitney *U* test was used for group comparison, and the Wilcoxon test for repeated measures. Linear correlations were tested using the Spearman rank correlation test. For the raw variables, in the case of group comparisons the chi-square test and the McNemar test were used for repeated measures. Statistical significance was set at *p* < 0.05.

## Results

**Fig. 1:**
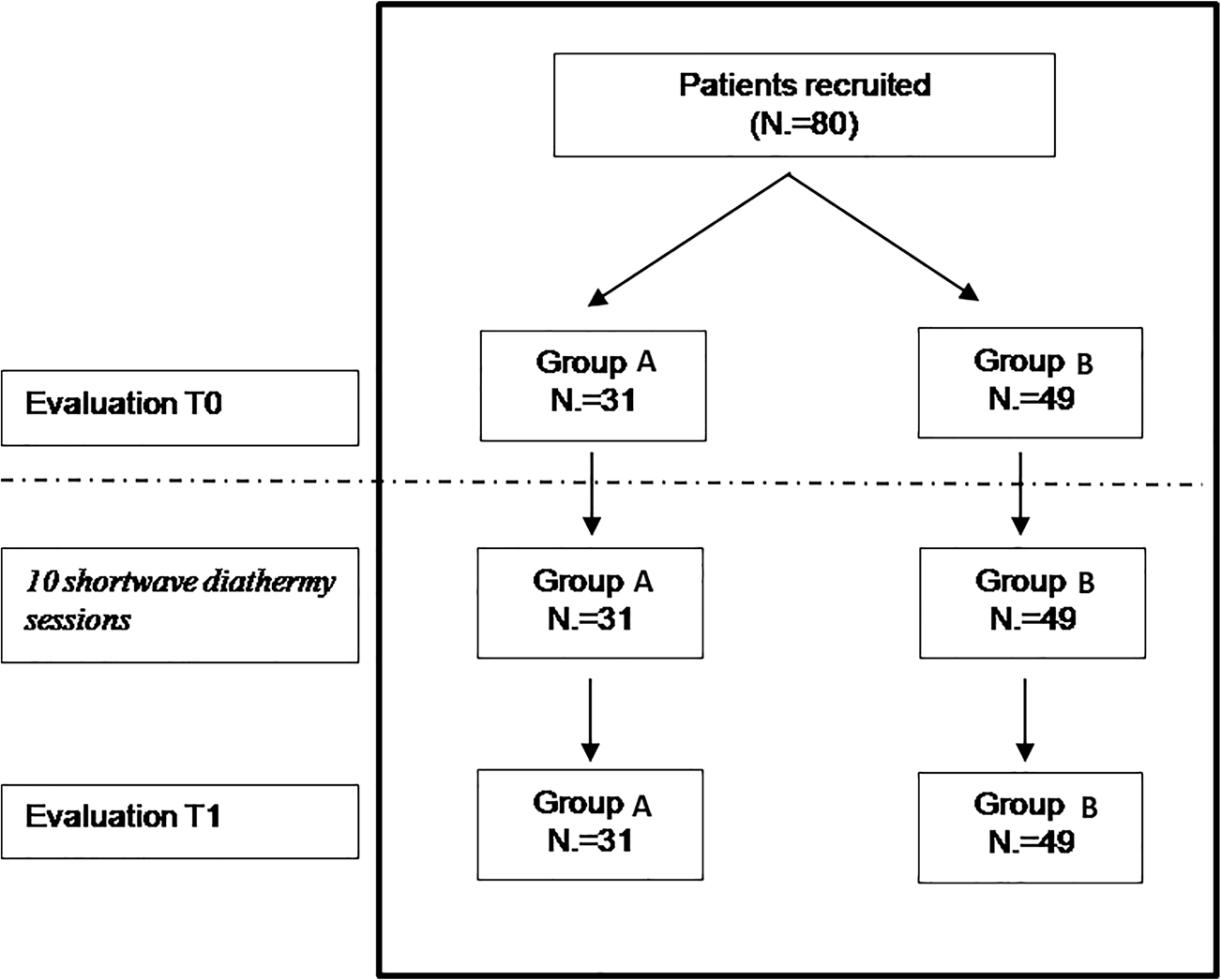
The overall plan of the study

**Fig. 2:**
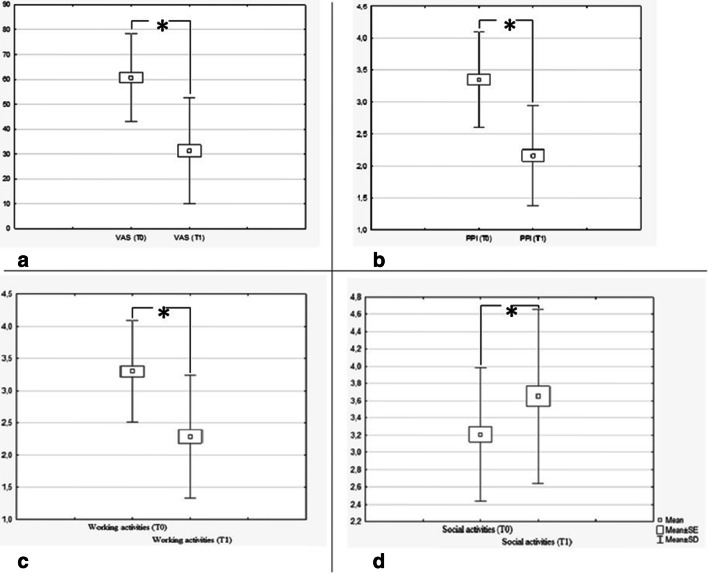
Graphical representation of comparison between T0 and T1 of the visual analog scale (VAS) (**a**), present pain intensity (PPI) (**b**), pain in work-related activity limitations (**c**), and in non-work-related (social activities) (**d**). All measures improved at T1. *p < 0.01

**Fig. 3:**
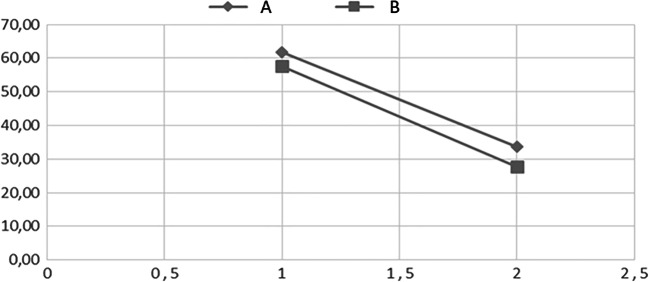
Visual analogic scale before treatment (T0) and after SWD treatment (T1). Group A, neck or low back pain with or without neck or back pain with or without radicular irradiation of pain; group B, nociceptive pain

**Fig. 4:**
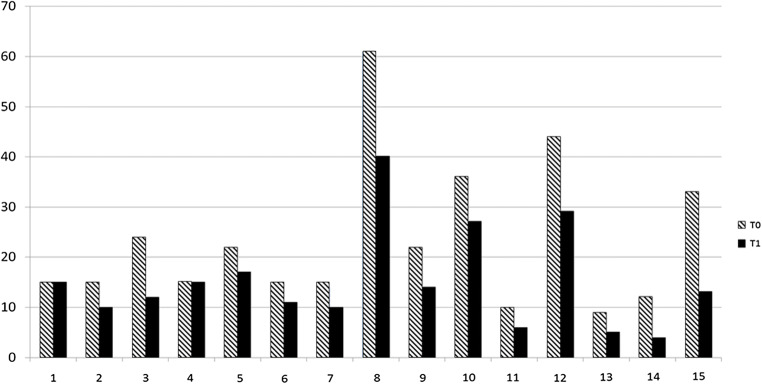
Absolute frequencies of each type of pain at T0 and T1. 1, throbbing; 2, shooting; 3, stabbing; 4, sharp; 5, cramping; 6, gnawing; 7, hot/burning; 8, aching; 9, heavy; 10, tender; 11, splitting; 12, tiring/exhausting; 13, sickening; 14, fearful; and 15, punishing/cruel

The study’s flow chart is reported in Fig. 1. Eighty participants (M = 31) were enrolled with mean age at the first evaluation 56 ± 12.49 years. All subjects were re-evaluated after an average of 55.74 days from the first evaluation (± 7.6 days, median = 54, range 52–62). Group A (both nociceptive and neuropathic pain) included 31 subjects (M = 13, mean age 60.4 years, range 43.3–80.8). Group B (nociceptive pain) included 49 subjects (M = 18, mean age 53.3 years, range 26.8–81.6). There were no dropouts from the study. Table 1 reports VAS and PPI values in the 2 groups at time T0 and T1. A significant improvement in VAS at T1 versus T0 (*p* < 0.01) emerged (Fig. 2a). A statistically significant decrease of PPI score at T1 (*p* < 0.01) was detected (Fig. 2b). The evaluation of in- and outdoor work-related activity limitations showed a statistically significant decrease between T1 and T0 (*p* < 0.01) (Fig. 2c). The limitations caused by pain in non-work-related activities diminished at T1 vs T0, (*p* < 0.01) as shown in Fig. 2d. VAS score decreased between T0 and T1 in each group, but no significant difference among the two groups was detected (Fig. 3). The average number of pain symptoms, as reported in the McGill questionnaire, decreased at T1 (2.8) compared to T0 (4.32) (*p* < 0.01). Frequency and the intensity of pain decreased from T0 to T1 (Fig. 4). No adverse effects or events were reported during the study.

**Table 1:** Mean value of visual analogic scale (expressed in mm) and present pain intensity index (PPI) and minimum and maximum values in two groups

Group	Number of subjects	VAS (T0) mean (min and max values)	VAS (T1) mean (min and max values)	*p* value T1 vs T0	Present pain intensity index (PPI) (T0) mean (min and max values)	Present pain intensity index (PPI) (T1) mean (min and max values)	*p* value T1 vs T0
A	31	57.55 (22, 86)	27.68 (0, 68)	< 0.01	3.32 (2, 5)	1.97 (0, 3)	< 0.01
B	49	62.76 (21, 100)	33.63 (0, 68)	< 0.01	3.37 (2, 6)	2.29 (1, 4)	< 0.01

A, group with both nociceptive and neuropathic pain; B, group with nociceptive pain. There was no significant difference in VAS score and age between the two groups at time T0

## Discussion

We investigated the efficacy of continuous SWD administered according to personalized paradigms to treat musculoskeletal pain. A consistent pain reduction and a marked improvement of quality of life were reported by our cohort.

The peculiar characteristic of this physical agent, which is effective in heating deep tissues without a risk of superficial hyperheating, and the possibility to modulate wave frequencies according to individual clinical requirements, makes this tool an effective therapeutic option in musculoskeletal pain. Physical effects of hyperthermia have been demonstrated: here we provide evidence of the potential to tailor these effects based on the different frequency penetration.

SWD has been prescribed for various medical conditions since the early twentieth century (McCarthy et al. 2006). Most of previously published research focused on pSWD, which consists in intermittent trains of high-frequency radio waves. The intermittent stimulation paradigm rests on the substantial reduction of risk of burns, which in turn reduces the overall heating effects. Individually tailored paradigms of cSWD aim at overcoming this limit. Several studies reported that pSWD has a long-term efficacy in osteoarthritis (OA) both at low (14.5 W power, 19 min treatment duration, and 17 kJ total energy) and high doses (14.5 W power, 38-min treatment duration, and 33 kJ total energy) (Fukuda et al. 2011). SWD associated with isokinetic exercise augments performance, reduces pain, and improves function in OA (Cetin et al. 2008). Eight hertz pSWD provides remarkable pain relief and improves daily quality of life in OA (Takahashi et al. 2009). pSWD was reported to be effective also for treatment of chronic low back pain (Ahmed et al. 2009) and in entrapment neuropathies, such as carpal tunnel syndrome (Incebiyik et al. 2015). The combination of thermal pulsed short-wave diathermy and joint mobilizations was effective in restoring active range of motion (ROM) of elbow extension in patients who lacked full ROM after injury or surgery (Draper 2014).

In fact, not all published studies confirm pSWD efficacy (McCarthy et al. 2006; Laufer and Dar 2012). Ultrasound therapy and SWD apparently did not provide different functional outcomes in knee OA in 61 women (Jan and Lai 1991). The 2017 American College of Physicians (ACP) guideline on noninvasive treatment of low back pain concluded that evidence is insufficient to determine the effectiveness of SWD (Qaseem et al. 2017).

These contrasting results point to the difficulty of obtaining homogeneous data from well-designed studies and call for the scientific community to address this issue, in light of the low-cost, ease of use of SWD. The main problems are high variability in outcome measures, inconsistencies in reporting the treatment dosage, high variability in treatment protocols, and lack of long-term follow-up studies (Laufer and Dar 2012). In fact, the intensity and duration of hyperthermia used in a clinical setting are determined on the basis of experience (Takahashi et al. 2009), because there are no standardized protocols for SWD treatment. Another possible cause of confusion is the degree of thermal sensation. In a meta-analysis (Laufer and Dar 2012), significant effects on pain and performance are found only when SWD evoked a local thermal sensation (local thermal effect). Unfortunately, few studies reported whether or not a thermal sensation was induced and often different definitions of thermal sensation were reported. This is a crucial point because the thermal effects are essential for therapeutic results, with effectiveness likely related to the temperature increase. Heat must penetrate deep tissues and be maintained constant for a given interval of time. When correctly administered, high and deep temperatures are reached rapidly and for long time, with low risk of burns. An increase of 4 °C has been reported as necessary to increase collagen extensibility and inhibit sympathetic activity (Lehmann et al. 1970). Continuous SWD (8 MHz, 200 W for 20 min) showed an increase of intraarticular temperature from 34.4 to 39.4 °C.

In our cohort, we believe that the main effect of pain reduction, which in turn has an impact on quality of life, was related to the well-known physical effects of heat. Increased vasodilatation and efflux from the affected tissue of pain mediators (Goats 1989), associated with the muscle-relaxant effect of heat, may be the main contributors to nociceptive pain reduction. The neuropathic component of pain may be mediated by the so-called gait control mechanism, in which inhibition of nociceptive transmission by of A-alpha and A-beta fibers (Melzack et al. 1977) or stimulation of cutaneous thermoreceptors may temporarily block pain information transmission at the spinal cord (Goats 1989).

The device we deployed in the present protocol allows a modulation of frequencies: this unique feature permits a tailored therapy based on the target anatomical structure and on individual burning pain thresholds. To treat more superficial structures, i.e., tendons and nerves, lower frequencies were applied, whereas for joints or deep nerves, e.g., knee, higher frequencies were selected. The extreme flexibility of the treatment protocol permits a personalization of the physical agent therapy, which is likely to have strongly contributed to the high rate of pain reduction in our cohort.

There is an overall neglect of the scientific community for the use of SWD in musculoskeletal disorders, the only exception being osteoarthritis. Our data support the effectiveness of cSWD for a wide range of musculoskeletal-related pain. In many studies, SWD was associated with kinesiotherapy (Cetin et al. 2008; Fukuda et al. 2011), which could positively influence the results. The efficacy of our protocol, which did not associate therapeutical exercise, points to the strong positive effect of personalized targeted cSWD. Its efficacy is likely related to the flexibility of delivery, which allows adjusting heat penetration according to the affected structures (tendons, muscles, bony parts) and modulating it based on individual response. Previous studies applied non-variable protocols in terms of frequency and power, which could give reason of the high positive responses in our cohort. In conclusion, continuous personalized SWD is effective and safe for treating of pain in patients with musculoskeletal disorders.

## Limitations

The limits of our study are related to the short follow-up period and to the absence of a control group. Short follow-up (4 weeks after end of treatment), while being proof of the efficacy of treatment, does not confirm the resolution of pathology, which in many osteo-muscular disorders may become chronic. Future studies using personalized SWD should address this issue, with the last follow-up at least after 6 months. The other main limitation of the study is the lack of a control group: generally, osteo-muscular disorders develop acutely, with only a small percentage developing the chronic form, i.e., 20% of acute pain becomes chronic (https://www.ninds.nih.gov/disorders/patient-caregiver-education/fact-sheets/low-back-pain-fact-sheet). Some disorders may even be self-limiting. Future studies need to address how SWD impacts on timing and quantification of pain reduction and functional restoration over time both in acute and in chronic conditions.

## Conclusions

The results of our study confirm that SWD is effective and safe and improves pain and quality of life in people with musculoskeletal disorders. However, these results cannot be considered conclusive. Further randomized, controlled clinical trials are necessary to determine the effectiveness of SWD and improve therapeutic protocols.
